# The role of Mitochondrial Fission Proteins in Mitochondrial Dynamics in Kidney Disease

**DOI:** 10.3390/ijms232314725

**Published:** 2022-11-25

**Authors:** Lingyu Qin, Shuhua Xi

**Affiliations:** Department of Environmental Health, School of Public Health, China Medical University, No. 77 Puhe Road, Shenyang North New Area, Shenyang 110122, China

**Keywords:** mitochondria, fission, fusion, post-translational modifications, kidney

## Abstract

Mitochondria have many forms and can change their shape through fusion and fission of the outer and inner membranes, called “mitochondrial dynamics”. Mitochondrial outer membrane proteins, such as mitochondrial fission protein 1 (FIS1), mitochondrial fission factor (MFF), mitochondrial 98 dynamics proteins of 49 kDa (MiD49), and mitochondrial dynamics proteins of 51 kDa (MiD51), can aggregate at the outer mitochondrial membrane and thus attract Dynamin-related protein 1 (DRP1) from the cytoplasm to the outer mitochondrial membrane, where DRP1 can perform a scissor-like function to cut a complete mitochondrion into two separate mitochondria. Other organelles can promote mitochondrial fission alongside mitochondria. FIS1 plays an important role in mitochondrial–lysosomal contacts, differentiating itself from other mitochondrial-fission-associated proteins. The contact between the two can also induce asymmetric mitochondrial fission. The kidney is a mitochondria-rich organ, requiring large amounts of mitochondria to produce energy for blood circulation and waste elimination. Pathological increases in mitochondrial fission can lead to kidney damage that can be ameliorated by suppressing their excessive fission. This article reviews the current knowledge on the key role of mitochondrial-fission-associated proteins in the pathogenesis of kidney injury and the role of their various post-translational modifications in activation or degradation of fission-associated proteins and targeted drug therapy.

## 1. Introduction

Mitochondria are the main site of respiration, producing ATP through oxidative phosphorylation (OXPHOS) to supply cells and organisms with vital activities [1]. Additionally, mitochondria have demonstrated excellent immunological effects against bacterial infections and viral infections. In past decades, research has been focused on mitochondrial bioenergetics, but, in recent years, the complexity and versatility of mitochondrial activities have been realized [2]. Individual mitochondria exist in various forms, such as small spherical, shorter, and longer tubular forms. However, further studies reveal that their different morphologies can be inter-connected and that the mitochondrial network can be regulated by the binding and breaking of the outer and inner mitochondrial membranes, a process called “mitochondrial dynamics” [3]. Mitochondrial fission and fusion mechanisms produce new mitochondria while eliminating old, damaged, and irreparable ones [4]. Mitochondrial fission proteins FIS1 and DRP1 are also involved in the cell cycle and cytoskeleton, thereby triggering fission. In contrast, the mitochondrial fusion proteins MFN1/MFN2 also play an important role in cell proliferation. Promoting mitochondrial-fission-related protein expression increases mitochondrial network fragmentation, while increasing mitochondrial fission promotes cytochrome C release from mitochondria and thus increases apoptosis, while inhibiting mitochondrial-fission-related proteins reduces mitochondrial fission and inhibits apoptosis to promote cell survival [5,6].

The kidney is the second most energy-demanding organ in the body after the heart [7]. The kidney has a large energy demand, and it needs much energy to finish cleaning the metabolic waste from the body’s blood. Hence, its mitochondrial content ranks high among many organs. When kidneys develop a disease, the mitochondria also appear abnormal. The mitochondria appear fragmented in kidney cells of patients with diabetic nephropathy, and the mitochondrial dynamics associated with protein DRP1 are often in an activated state of phosphorylation, leading to an increase in mitochondrial fission [8]. However, when mitochondrial fission increases, the permeability of the outer mitochondrial membrane increases, resulting in the release of its contents and a decrease in mitochondrial function, which can cause kidney cell damage, and inhibiting the increase in mitochondrial fission can protect kidney cells [9,10].

## 2. Mitochondrial Dynamics

**Figure 1:**
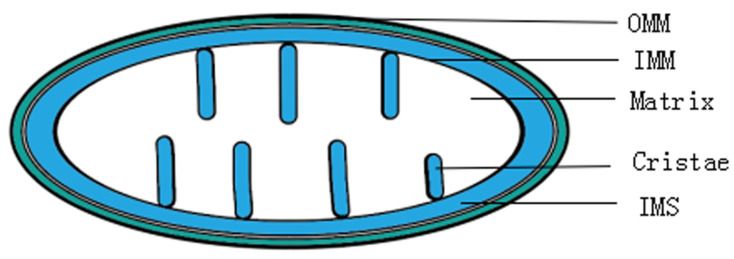
The structure of mitochondria. OMM: mitochondrial outer membrane. IMM: inner mitochondrial membrane. Cristae: mitochondrial inner membrane is depressed inward to form cristae. Matrix: internal space wrapped by the inner mitochondrial membrane. IMS: the space between the outer mitochondrial membrane and the inner mitochondrial membrane.

Mitochondria are powerful organelles, and the mitochondrial network plays an important function in the cell by providing energy, regulating programmed cell death, and producing reactive oxygen species (ROS) [11]. Mitochondria are composed of the outer mitochondrial membrane (OMM), inner mitochondrial membrane (IMM), cristae, matrix, and intermembrane space (IMS) (Figure 1). The OMM separates the mitochondria from the cell matrix, while the IMM forms cristae and can separate the mitochondrial matrix from the outer membrane interstitial region. In the past, mitochondria were often thought to exist in isolation, but they form highly dynamic mitochondrial networks interacting with each other. Furthermore, mitochondrial networks are not static, and the interaction between OMM and IMM of different mitochondria can change the mitochondrial network morphology [12,13]. While “mitochondrial dynamics” consists primarily of fission and fusion, many researchers include cristae remodeling in this category. It has been demonstrated that mitochondrial fission is commonly associated with mitochondrial dysfunction, whereas mitochondrial fusion can serve a protective function [14]. In cellular life activities, mitochondrial fusion and fission can occur in a short period, especially in the case of external-stress-induced fission or transient partial fusion events [15]. Fusion and fission are “double-edged swords” that are important for normal mitochondrial function. Mitochondrial fission produces small spherical mitochondria that play an important role in axonal cell transport, while mitochondrial fusion protects against external stimuli [16]. A decrease or increase in mitochondrial fission and fusion can lead to imbalances that affect mitochondrial function and ultimately lead to various diseases [17]. Normal cristae play an important role in cell metabolism and cell death, and reducing the width of cristae enhances ATP production and resistance to external death stimuli [18,19].

### 2.1. Mitochondrial Fission

**Figure 2:**
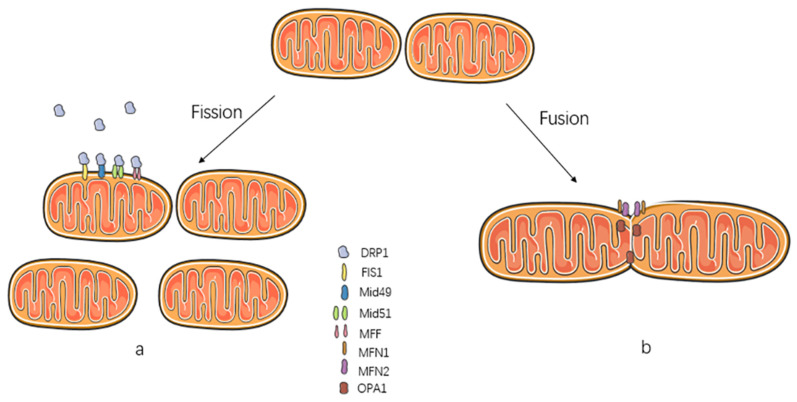
The process of mitochondrial fission and fusion. (**a**): The mitochondrial fission process and related proteins, mitochondrial outer membrane proteins FIS1, Mid49, Mid51, and MFF, are capable of recruiting DRP1 from the cytoplasm to the mitochondrial outer membrane. (**b**): The mitochondrial fusion process and related proteins, the mitochondrial outer membrane proteins MFN1 and MFN2, are responsible for the fusion of the outer mitochondrial membrane, and the mitochondrial inner membrane protein OPA1 is responsible for the fusion of the inner mitochondrial membrane.

Mitochondrial fission is the splitting of an intact mitochondrion into two separate mitochondria. Multiple factors, including the endoplasmic reticulum, lysosomes, Golgi-derived vesicles, and actin filaments, regulate mitochondrial fission [20,21,22,23,24]. Mitochondrial fission begins with labeling fission sites, usually the contact sites of the nucleoid markers of the mitochondrial matrix, the endoplasmic reticulum, and lysosomes [25,26]. Next, mitochondrial outer membrane protein proteins, including FIS1, MFF, MiD49, and MiD51, aggregate at a site in the mitochondrial outer membrane. The mitochondrial outer membrane protein receptor recruits the DRP1 from the cytoplasm to the site of mitochondrial fission (Figure 2). DRP1, a cytosolic dynamin GTPase, plays an important role in mitochondrial fission [27]. DRP1 can form oligomeric rings, and, as DRP1 aggregation increases, a hinge-like structure can form, severing OMM and IMM simultaneously [28]. FIS1 can reduce the number and viability of mitochondrial fusion proteins (MFN1, MFN2, and OPA1) by binding to them and fragmenting the mitochondrial network [29,30]. While it was thought that the inner and outer membranes were fissioned simultaneously during mitochondrial fission, new insights suggest that inner mitochondrial membrane fission is independent and increased short OPA1 isoform (S-OPA1) accumulation and translocated IMM protein mitochondrial protein 18 kDa (MTP18) expression can mediate IMM fission [28,31]. However, this latter view needs to be further tested.

### 2.2. Mitochondrial Fusion

Mitochondrial fusion refers to fusion of several mitochondria with intact structures into one longer mitochondrion. Mitochondrial fusion is divided into two parts, outer mitochondrial membrane fusion and inner membrane fusion. In the past, it was thought that the outer and inner mitochondrial membranes fused nearly simultaneously [32]. However, recent literature reports that IMM and OMM fusions are continuous, and mitochondrial fusion can be divided into “transient fusions” and “complete fusions”. Transient fusions typically interact obliquely or horizontally and, to a lesser extent, vertically; however, transient fusions tend to preserve the original mitochondrial morphology, whereas complete fusions are frequently longitudinal and capable of producing a complete mitochondrion [33]. MFN1 and MFN2 mediate outer membrane fusion in mammals (Figure 2) [34]. MFN1 and MFN2 proteins can interact between mitochondria, tethering two separate mitochondrial outer membranes via their HR2 structural domains [35,36]. This is the beginning of fission [37]. Moreover, MFN2 can act as a physical tether between mitochondria and the endoplasmic reticulum and regulate calcium ion signaling [38]. However, recent studies suggest that MFN2 has the opposite effect [39]. The specific role of MFN2 needs to be studied specifically in the context of different mechanisms of action in various cells. OPA1 plays an important role in IMM fusion [40]. OMA1 and AAA protease YME1L, mitochondrial proteases, can cleave OPA1 from the long (L-OPA1) to the short (S-OPA1) form. L-OPA1 is needed for mitochondrial fusion, while S-OPA1 is needed for mitochondrial fission [41]. OPA1 downregulation inhibits mitochondrial fusion, resulting in mitochondrial fragmentation [42]. Its upregulation can promote the fusion of mitochondria to protect cells via an anti-apoptotic effect. The use of drugs to increase OPA1 expression is of great research value for cytoprotection [43]. OPA1 can inhibit apoptosis independently of mitochondrial fusion and reduce cytochrome C release [44].

### 2.3. Cristae Remodeling

**Figure 3:**
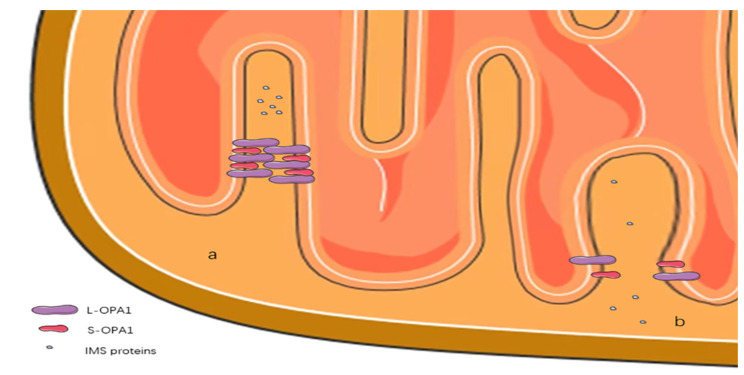
Cristae remodeling. (a): Under normal conditions, L-OPA1 and S-OPA1 are able to form oligomers to maintain the structure and function of the cristae. (b): The cristae remodel, the opening of the cristae becomes larger, and the contents of the cristae are released.

Cristae remodeling refers to changes in the length, width, abundance, and exit of the cristae in response to external stimuli [28]. Cristae morphology is related to the efficiency of energy production, apoptosis, and oxidative stress [45]. L-OPA1 and S-OPA1 can form oligomers that act as “gatekeepers” under normal conditions (Figure 3). When the cristae opening becomes larger and L-OPA1 decreases, cytochrome C that remains in the cristae is released, triggering a series of cell death behaviors [44].

### 2.4. Mitochondrial Dynamics with Other Cellular Organelles

#### 2.4.1. Endoplasmic Reticulum

**Figure 4:**
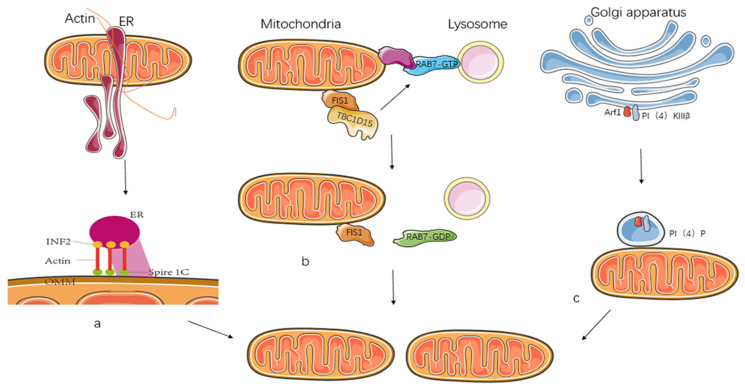
Interactions between mitochondria and other organelles promote mitochondrial fission. (**a**): The ER-bound INF2 and mitochondrial Spire1C induce actin nucleation and polymerization at mitochondria–ER contact sites. (**b**): The RAB7-GTP protein on the lysosome can bind to an unknown protein on the outer mitochondrial membrane to form a mitochondrial–lysosomal contact, and the mitochondrial outer membrane protein FIS1 is able to recruit TBC1D15, which is able to hydrolyze RAB7-GTP to RAB7-GDP and unbind it. (**c**): Golgi-derived vesicles containing PI(4)P contribute to mitochondrial division.

The mitochondria and endoplasmic reticulum (ER) can interact through protein–protein interactions, and the distance between them can be decreased to form contact sites (Figure 4). ER and mitochondria interaction is critical for calcium homeostasis and mitochondrial dynamics. At the ER–mitochondrial contact site, mitochondrial contraction followed by mitochondrial division has been observed [20]. ER tubules wrapped around the mitochondria can mark the site of DRP1 recruitment by the mitochondrial receptor; the assembled DRP1 aggregates and then forms a helix to contract the mitochondria; ER tubules play a localization role in mitochondrial division and even participate in mitochondrial fission. DRP1 cooperates with formin 2 (INF2) on the ER and the formin-binding protein spire 1C on the outer mitochondrial membrane, leading to actin accumulation before DRP1 recruitment to the fission site, thereby promoting mitochondrial precontraction [23]. The connection between the ER and the mitochondrial membrane results from the protein complex’s physical binding. The first is a complex between FIS1 on the mitochondrial membrane and BAP31 on the ER, frequently associated with cell death, and the second is that MFN2 also plays an important role in the contact between the two [46]. ER structures, particularly renal tubule ER–mitochondrial contacts, are necessary for steady-state mtDNA replication, and mtDNA-containing nucleoids are early markers of nascent mitochondrial division sites [47]. According to the literature, ORP1L-mediated PI(4)P signaling at the ER–lysosomal–mitochondrial three-way contact contributes to mitochondrial division [48].

#### 2.4.2. Lysosomes

Mitochondrial–lysosomal contacts are also critical and play an important role in several diseases. Various protein mechanisms on the mitochondrial and lysosomal membranes regulate mitochondrion–lysosome contact site tethering. It can function as a linkage through the binding of the RAB7GTP protein on the lysosomal surface and an unknown mitochondrial protein (Figure 4) [49]. FIS1 is a mitochondrial-fission-related outer mitochondrial membrane protein that recruits TBC1D15, the activator protein of RAB7GTP, to the mitochondria and induces RAB7GTP conversion on lysosomes to RAB7GDP, thereby unbinding the mitochondria from the lysosomes [21]. Lysosomal dissociation from the mitochondrial division site increases mitochondrial fission [49].

#### 2.4.3. Golgi

Mitochondrial dynamics are related to numerous organelles, and mitochondrial division is related to the ER, lysosomes, and Golgi. Arf proteins are small GTP-binding (G) proteins. Arf proteins are classified into three groups; Arf1 is highly conserved in evolutionary time and plays an important role in the structure and function of the Golgi [50]. Arf1 and PI(4)KIIIβ are mainly restricted to the Golgi apparatus, and Arf1 can activate PI(4)KIIIβ to produce PI(4)P, which is involved in late mitochondrial fission (Figure 4) [51].

#### 2.4.4. Actin

It has been demonstrated that mitochondrial fission requires preconstruction, which is inseparable from the actin cytoskeleton, and that the proteins involved, Spire1C and INF2, play an important role in inducing mitochondrial constriction (Figure 4) [52]. Spire1C is a protein on the OMM, and the ER-anchored junctional isoform of INF2 can promote mitochondrial constriction by regulating its actin polymerization activity. Spire1C interacts with INF2 to drive actin filament formation in narrowed mitochondria [24]. It can further promote the contact between mitochondria and ER and mitochondrial fission.

### 2.5. ROS in Mitochondrial Dynamics

ROS are small, highly reactive molecules that oxidize proteins, lipids, and DNA [53]. In mammals, mitochondria are the primary source of ROS. Additionally, ROS can promote mitochondrial fission by increasing mitochondrial fission proteins and inducing post-translational modifications to promote apoptosis [54,55,56]. Persistent mitochondrial fragmentation in cells has been observed to be frequently associated with elevated ROS [57,58]. ROS plays an important potential role in promoting mitochondrial fission. When external stimuli, such as hypoxia or drug interventions, cause elevated intracellular ROS, they frequently cause an increase in mitochondrial-fission-related proteins DRP1 and FIS1 and promote their post-translational modification to activate fission-related proteins, resulting in a pathological increase in mitochondrial fission that can have adverse effects on cells [59,60].

## 3. Structure and Function of Mitochondrial Fission Proteins FIS1/DRP1 and the Role of Post-Translational Modifications

Mitochondrial fission is not only ordinary monotonic fission; mitochondrial fission is divided into midzone and peripheral fission. Peripheral fission upstream displays signs of stress and damage that may lead to degradation, while midzone fission may contribute to biogenesis. Peripheral fission is inseparable from the role of FIS1 [61]. FIS1 deletion prevents apoptosis upstream of Bax translocation, whereas DRP1 deletion protects cells downstream of Bax translocation and upstream of cytochrome C release [32,62]. BAK and BAX can oligomerize on the OMM to drive its rupture. Subsequent increases in mitochondrial outer membrane permeability (MOMP) lead to outflow of pro-apoptotic factors (including cytochrome C and mtDNA) into the cytoplasmic matrix, triggering various programmed cell deaths. In addition to causing mitochondrial fission, FIS1/DRP1 interaction has been demonstrated to cause mitochondrial dysfunction in cells, such as decreased mitochondrial membrane potential, oxidative stress, and bioenergetic failure. When inhibitors that inhibit the interaction between the two are used, mitochondrial dysfunction can be effectively improved [63]. This indicates that DRP1 and FIS1 play a significant role in mitochondrial dynamics and apoptosis. Protein post-translational modifications (PTMs) occur when enzymes catalyze one or more amino acid residues of a protein to increase or decrease a chemical group, resulting in the protein’s degradation or activation. Popularly speaking, post-translation modification refers to chemical modification of proteins after translation. Only the structures of the mitochondrial fission proteins FIS1 and DRP1 and the role played by post-translational modifications in the regulation of protein function are highlighted here.

### 3.1. FIS1

#### 3.1.1. Structure and Function of FIS1

**Figure 5:**
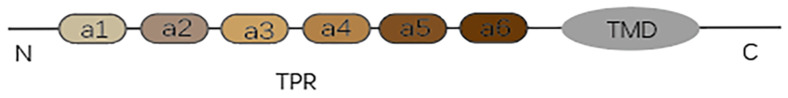
Schematic diagram of the FIS1 structure. TPR, a1–a6 are six a-helices connected by short rings; residues Val11-Ala27, Lys32-Val43, Asn48-Leu62, Lys67-Arg83, Tyr87-Thr100, Asn105-Lys120 form α1, α2, α3, α4, α5, α6, respectively. The TMD region is responsible for the location of FIS1.

FIS1 has a flexible N-terminal tail, six a-helices connected by short loops to form a core domain structurally similar to a tridecapeptide repeat (TPR), and a disordered C-terminus containing a transmembrane region (TMD) (Figure 5) [64,65]. According to studies, the N-terminal structure of FIS1 is important for its mitochondrial fission activity [66]. The TPR-like structural domain is a helix-rotation helix pattern that is usually organized into a tandem structure capable of mediating protein–protein interactions that are critical to its biological function [67]. While the TMD plays a role in transporting the protein to the outer mitochondrial membrane, without this region, FIS1 would not be able to localize to the mitochondria and diffuse in the cytoplasmic region [64].

FIS1 can recruit DRP1 to the OMM to promote mitochondrial fission [63,68,69,70,71,72]. However, recent research discovered that mitochondria could regulate contact with lysosomes through FIS1 to trigger asymmetric mitochondrial division [61,73]. FIS1 overexpression can lead to mitochondrial fragmentation during acute kidney injury [74]. The same can happen in children with membranous nephropathy [75]. High expression of FIS1 promotes mitochondrial division, apoptosis, and pyroptosis of cells, thus inducing nephrotoxicity [76]. However, it has also been shown that FIS1 also plays an important role in mitochondrial autophagy and can play a protective role in diabetic nephropathy by participating in mitochondrial quality control through the adaptive mitochondrial autophagy pathway [77].

#### 3.1.2. Post-Translational Modifications of FIS1 during Mitochondrial Fission

FIS1 is mainly regulated by several post-translational modifications, mainly including: phosphorylation, ubiquitination, SUMOylation, and acetylation (Table 1). These modifications play an important role in activation and degradation of FIS1.

##### Phosphorylation

Phosphorylation is widespread post-translational modification of proteins and plays an important role in regulating protein and cellular functions. Although numerous phosphorylation sites have been identified, this is only the tip of the iceberg; more sites remain to be discovered [78]. Phosphorylation is most common at serine, followed by threonine and tyrosine, which occur at a frequency of approximately 11.2:2.5:1 but can also occur at other additional sites [79]. FIS1 can be directly phosphorylated at the tyrosine 38 site (Tyr38) by other factors, such as MET, and FIS1 pY38 can recruit Drp1 to promote mitochondrial fission, resulting in fragmented mitochondrial networks [68]. Studies have demonstrated that DNA-dependent protein kinase catalytic subunit (DNA-PKcs) can phosphorylate Fis1 [80]. Phosphorylation on FIS1 promotes mitochondrial fission. In the case of increased pathological mitochondrial fission due to kidney injury, phosphorylation of FIS1 can be inhibited to play a role in mitigating injury and protection, introducing a new idea of therapeutic protection.

##### Ubiquitination

Ubiquitin (Ub) is a relatively conserved protein that is often associated with protein degradation [81]. Ubiquitin is a 76-amino-acid protein and, therefore, has many potential sites for additional post-translational modifications [82]. Ubiquitination covalently couples to lysine residues on target proteins through a series of enzymatic reactions that activate (E1), conjugate (E2), and ligate (E3) enzymes. Parkin is an E3 ubiquitin ligase that regulates ubiquitination of its substrates and can mediate protein degradation [83]. PINK can regulate Parkin activity by regulating Parkin phosphorylation, so PINK/Parkin is often used in combination to regulate protein activity in mitochondria [84,85]. FIS1 is one of the targets of Parkin-mediated ubiquitination and is able to degrade FIS1 [86]. It has also been shown that MARCH5/MITOL (E3 ligase) can influence mitochondrial morphology by affecting FIS1 [87,88].

##### SUMOylation

Sentrin/small-ubiquitin-related modifier (SUMO) can regulate protein modification pathways for a variety of biological processes, such as cell division, DNA replication and repair, cell death, and cellular metabolism. SUMOylation works somewhat similar to ubiquitination, which is why SUMOization is also called mini-ubiquitination. However, the enzymes involved in the two are completely different. Another difference between the two is that ubiquitination is relatively complex, while the biochemical reactions of SUMOization and de-SUMOization are relatively mild [89]. SUMO couples to lysine residues in target proteins via SUMO-specific activating (E1), conjugating (E2), and ligating (E3) enzyme-catalyzed isopeptide bonds. SUMOylation is a reversible process and can be de-SUMOylated by the activity of the SENP protease family [90]. The lysine 149 site (K149) of FIS1 can be SUMOized. Blocking SUMOylation of Fis1 enhances Fis1 mitochondrial localization [91]. SENP3, a member of the SENP protease family, is a de-SUMOized enzyme that de-SUMO-izes FIS1, thereby enhancing its mitochondrial targeting.

##### Acetylation

Lysine acetylation (acetylation) is a reversible post-translational modification that plays an essential role as a player in the life processes of many cells. Acetylation is mainly regulated by lysine acetyltransferases (KATs) and lysine deacetylases (HDAC). Acetylation is widespread, and the acetylation status of many sites is controlled by the enzymatic activity of NAD-dependent deacetylase Sirtuin 3 (SIRT3). SIRT3 is the only mitochondrial member with strong deacetylation activity; SIRT3 is the major NAD-dependent lysine deacetylase in mitochondria, with many acetylation sites in mitochondrial proteins that are involved in mitochondrial dynamics, ATP production, pyruvate metabolism, and antioxidant defense [92,93,94]. SIRT3 plays an important role in mitochondrial fission, and knockdown of SIRT3 increases FIS1 expression and increases mitochondrial fragmentation [95,96]. SIRT3 is essential for maintaining the balance between mitochondrial fission and fusion.

### 3.2. DRP1

#### 3.2.1. Structure and Function of DRP1

**Figure 6:**
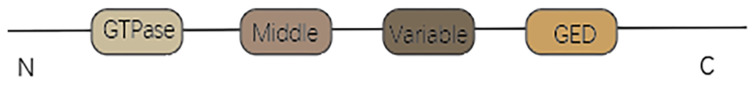
Schematic diagram of the DRP1 structure. GTPase: highly conserved N-terminal GTPase domain. Middle: helical middle domain. Variable: variable domain (also known as insert B). GED: a C-terminal GTPase effector domain.

DRP1 is a cytoplasmic guanosine triphosphatase that is activated and can be recruited from the cytoplasm to the OMM by mitochondrial outer membrane proteins. DRP1 protein is known to have four structural domains: the C-terminal GTPase effector domain (GED), the variable structural domain (variable), the helical intermediate structural domain (middle), and the highly conserved N-terminal GTPase structural domain (GTPase) (Figure 6). The DRP1 protein is recruited to the membrane by the receptor protein on the OMM and then severs the inner and outer mitochondrial membranes by forming a dimer or tetramer through a variable structural domain and binding to the outer mitochondrial membrane to act as a hinge [97].

DRP1 promotes mitochondrial fission, increased expression of DRP1 promotes fragmentation of the mitochondrial network, while inhibition of DRP1 expression can lead to highly elongated mitochondria [98]. In diabetic nephropathy, mitochondrial fission can be reduced by decreasing DRP1 expression to stop progression of diabetic nephropathy [99]. Inhibition of DRP1 plays an important protective role in protection against ischemic acute kidney injury [100].

#### 3.2.2. Post-Translational Modifications of DRP1 during Mitochondrial Fission

DRP1 is mainly regulated by the following post-translational modifications: phosphorylation, ubiquitination, SUMOylation, O-GlcNAcylation, acetylation, S-Nitrosylation, etc (Table 2). These modifications mainly occur in the variable structural domain and the C-terminal GTPase effector structural domain.

##### Phosphorylation

There are usually many phosphorylation sites of DRP1, such as serine sites: Ser-40, Ser-44, Ser-579, Ser-585, Ser-592, Ser-616, Ser-637, Ser-656, and Ser-693 [101]. The most studied DRP1 phosphorylation sites are Ser-616 and Ser-637. Activation of the Ser-616 site of DRP1 promotes DRP1 movement from the cytoplasm to the OMM, interacting with OMM proteins and thus promoting mitochondrial fission [102]. There are many proteins that can activate the Ser-616 site of DRP1, such as Stat2, Rho-associated protein kinase (ROCK), PKCδ, ERK1/2, calmodulin-dependent protein kinase II (CaMKII), and Cdk5 [103,104,105,106,107,108,109]. In contrast, the Ser-637 and Ser-616 sites of DRP1 have opposite effects. Ser-637 plays an inhibitory role on DRP1, and inactivation of DRP1 inhibits DRP1 translocation to the OMM, thus reducing mitochondrial fission [110,111]. The Ser-637 site of DRP1 is normally regulated by, PKA, PKD, CaN, and AMPK [112].

##### Ubiquitination

Inactivation of Parkin often leads to reduced degradation of Drp1, resulting in increased translocation of Drp1 activity, leading to excessive mitochondrial division and ultimately to development of certain diseases [101]. MRCH5 is an OMM-related E3 ubiquitin ligase that controls mitochondrial function [113]. According to the literature, MARCH5 plays a crucial role in controlling mitochondrial morphology by regulating Drp1 activity [114]. Some studies have demonstrated that SQSTM1/P62 has the same effect on DRP1 [115,116].

##### SUMOization

DRP1 is SUMO-ized, which causes the modified DRP1 to be released back into the cytoplasm and inhibits mitochondrial division, while the de-SUMOized protein SENP3 de-SUMOizes DRP1, which, in turn, restores DRP1 function, promotes DRP1 translocation to the OMM, increases the time DRP1 spends on the OMM, and thus promotes mitochondrial division, cytochrome C release, and cell death [117,118,119]. Dual-specificity phosphatase 6 (DUSP6) is a dephosphorylase. DUSP6 can dephosphorylate DRP1 Ser-616, and the K234 site of DUSP6 itself can be modified by SUMOization. DUSP6 can become stable after being modified [120].

##### O-GlcNAcylation

Protein glycosylation is the covalent attachment of monosaccharides or glycans to select residues of the target protein, and glycosylation occurs mainly on serine and threonine residues [121]. O-GlcNAcylation is mainly regulated by two proteins, OGT and OGA. The OGT catalyzes the O-linked N-acetylglucosamine (O-GlcNAc), while OGA removes the O-GlcNAc residue. Threonine residues on DRP1, such as Thr-585 and Thr-586, are able to undergo glycosylation [122]. The ability to promote DRP1 glycosylation by inhibiting OGA has been demonstrated to promote its mitochondrial fragmentation and damage cells [121].

##### Acetylation

DRP1 is able to be acetylated at K642. K642 is located within GED, which regulates DRP1 oligomerization and GTPase activity. K642 acetylation increases the stability of DRP1, and Drp1 acetylation at K642 promotes phosphorylation of Drp1 at S616, thereby promoting mitochondrial fission [123].

##### S-Nitrosylation

S-Nitrosylation is a post-translational modification of the free sulfhydryl group in cysteine by nitric oxide, a process that is reversible, and nitric oxide is able to covalently bind to the target protein to form S-nitrosothiol (SNO) [124]. Many proteins in mammals are S-Nitrosylated. S-Nitrosylation plays an important role in cell proliferation, apoptosis, cell migration, and the tumor microenvironment [125,126,127]. DRP1 is able to be S-Nitrosylated at the 644 site of cysteine (Cys644), and the modified DRP1 is overactivated with increased translocation, promoting mitochondrial fission and fragmentation of the mitochondrial network, ultimately leading to cell death [128,129]. It has also been shown that S-Nitrosylation of DRP1 may regulate DRP1-S616 phosphorylation, thereby promoting mitochondrial fission [130].

## 4. FIS1- and DRP1-Dependent Mitochondrial Dynamics in Kidney Disease

### 4.1. Acute Kidney Injury (AKI)

AKI is now considered to be a public health problem with a very high human impact and, to some extent, can contribute to progression of chronic kidney disease (CKD) [131]. AKI is a frequent complication in hospitalized patients [132] and exhibits high mortality and high prevalence. AKI is characterized by a strong inflammatory response in the kidneys and body circulation, and this systemic inflammatory response leads to dysfunction of other organs, in addition to often causing acidosis, electrolyte abnormalities, and infections [133]. AKI is often characterized by an increase in serum creatinine and blood urea nitrogen and a decrease in urine output [134]. In addition, if the tubular recovery is incomplete in AKI, it can lead to long-term dysfunction and, to a certain extent, can evolve into CKD. During AKI, mitochondria are often fragmented, and mitochondrial fission proteins, such as FIS1 and DRP1, show an increase. Therefore, drugs can inhibit the increase in mitochondrial fission proteins, improve the fragmentation of mitochondria, and promote the fusion of mitochondria [135]. Increased mitochondrial fission induces mitochondrial dysfunction, leading to altered renal cell function and structure, as well as loss of renal function. It is possible to restore renal function by improving mitochondrial homeostasis and function. It is expected that DRP1 and FIS1 inhibitors may restore mitochondrial and renal function in AKI models [7]. AKI is accompanied by excessive ROS production, promoting mitochondrial fission protein expression and activation, causing renal tubular cell injury, apoptosis, and necrosis [136]. Studies have indicated that drugs that eliminate ROS, such as quercetin and resveratrol, can effectively alleviate acute kidney injury [137,138].

### 4.2. Chronic Kidney Disease (CKD)

CKD is a relatively common disease, with a prevalence of about 10.6–13.4%. The prevalence has risen during recent decades: it shows a trend that CKD progresses less in women than in men [139]. CKD often brings about vascular endothelial dysfunction, and cardiovascular disease is the leading cause of death in CKD patients [140]. In contrast, studies have demonstrated that aortic endothelial cells in CKD mice exhibit increased DRP1 expression and decreased MFN2 levels, and that pharmacological inhibition of DRP1 expression improves endothelial dysfunction [141,142]. Chronic renal failure (CRF), characterized by glomerulosclerosis and interstitial fibrosis, is a common end stage of various chronic kidney diseases (CKDs). TGF-β1 is an early biomarker of fibrosis. Downregulation of mitochondrial fusion proteins OPA1 and MFN2 and upregulation of fission protein DRP1 are often observed in cells with high TGF-β1 expression. The imbalance of mitochondrial dynamics plays an important role in tissue pathogenesis, so it is of great interest to block the effect of TGF-β1 on mitochondrial-dynamics-related proteins by drugs [143]. During progression of AKI to CKD, there is often an increase in mitochondrial fission and a decrease in mitochondrial fusion, and disturbances in mitochondrial dynamics promote development of AKI to CKD. When DRP1 and FIS1 are reduced, progression of AKI to CKD can be effectively mitigated [95]. It is documented that mitochondrial dysfunction may affect the severity of CKD, and persistent mitochondrial dysfunction leads to persistent tubular damage. This may also affect the kidney’s recovery from AKI and further progression to CKD [144]. Since increased FIS1/DRP1 expression in CKD can cause subsequent mitochondrial dysfunction, inhibiting FIS1/DRP1 expression is critical in improving and preventing CKD. Increasing evidence indicates that elevated ROS levels play a major role in CKD pathogenesis. Elevated intracellular ROS levels can cause lipid, DNA, and protein oxidation, as well as mitochondrial fragmentation, resulting in cellular damage [145]. Therefore, pharmacological treatment to reduce ROS is also required to improve CKD.

### 4.3. Diabetic Kidney Disease (DKD)

DKD has become the major cause of chronic kidney disease worldwide, and the rise in the prevalence of diabetic nephropathy is similar to the dramatic increase in the global prevalence of diabetes [146,147]. Diabetic nephropathy is the strongest predictor of mortality in diabetic patients [148]. Renal tubular injury is an early feature of DKD; renal tubular cells have abundant mitochondria. Mitochondrial fragmentation is an important pathogenic feature of renal tubular cell injury, and FIS1 and DRP1 play an important role in mitochondrial fission. In renal tubular cells with DKD, mitochondria are fragmented and FIS1 is increasing, while downregulation of FIS1 can improve mitochondrial morphology and reduce apoptosis of renal cells [149,150]. It has been shown that pharmacological inhibition of DRP1 expression and protection of mitochondria from excessive division can effectively stop progression of DKD [99]. DKD is accompanied by mitochondrial dysfunction, a decrease in renal function due to tubular damage, and mitochondrial dysfunction contributes to DKD progression and development [151]. When FIS1/DRP1 is decreased, increasing MFN2, a mitochondrial-fusion-related protein, can improve mitochondrial dysfunction [152]. Diabetic nephropathy models exhibit high ROS, which induces inflammation, fibrosis, and activation of mitochondrial fission proteins. Berberine (BBR) can effectively reduce ROS and DRP1 expression to protect the foot cells in diabetic nephropathy [99].

## 5. Drugs Targeting FIS1 and DRP1 and Their Role in Kidney Disease

FIS1 and DRP1 play pivotal roles in mitochondrial fission. Therefore, using drugs targeting FIS1 and DRP1 is important in improving kidney disease. Cisplatin is a chemotherapeutic agent used in treatment of cancer, but its use is often accompanied by AKI [153]. Meanwhile, curcumin can target FIS1 and prevent elevation of FIS1 as well as prevent decrease in mitochondrial fusion protein; the protective and preventive role of curcumin in cisplatin-induced AKI cannot be ignored [135]. Emodin is an anthraquinone derivative that significantly prevents increase in DRP1 expression and, thus, promotes reduced renal cell injury in AKI [154], and magnesium stigmasterate B plays a similar protective role in AKI by targeting DRP1 to renal cells [155]. High glucose often causes fragmentation of the mitochondrial network and cell death. Empagliflozin is a drug that slows progression of renal disease in diabetic patients. It was shown that, in the presence of high glucose, 100 nM of empagliflozin was effective in reducing the expression of DRP1 and 500 nM of empagliflozin was effective in reducing the expression of FIS1, in addition to the upregulation of mitochondrial fusion proteins and the downregulation of ROS exhibited by empagliflozin [156]. BBR is an isoquinoline alkaloid found in Chinese herbal medicines and widely used in treatment of diabetes. BBR protects renal cells by targeting DRP1 and inhibiting DRP1-mediated mitochondrial fission [99]. Mdivi1, a mitochondrial fission inhibitor, inhibits DRP1 expression and is effective in improving and preventing renal fibrosis after Mdivi1 administration [157], and has cytoprotective effects on renal epithelial cells in an animal model of acute kidney injury [158]. Polydatin (PD) is a resveratrol glycoside that protects kidney function in patients with diabetic nephropathy, and PD is able to inhibit podocyte injury by suppressing DRP1 expression and mitochondrial fragmentation [159]. Formononetin (FMN) is a novel isoflavonoid constituent isolated from Astragalus membranaceus; it can reduce mitochondrial fission protein, increase mitochondrial fusion protein, and improve mitochondrial damage in diabetic nephropathy [150]. P110 is an inhibitor of DRP1 and FIS1 interaction and can improve mitochondrial damage by blocking the interaction between the two fissions [160].

## 6. Summary and Outlook

Mitochondria are organelles with diverse roles and are involved in a variety of biological functions. Mitochondria can regulate other organelles, and, in turn, other organelles can regulate the morphological functions of mitochondria. In the past, attention was often focused on the energetics of mitochondria, but, as research progressed, it became clear that the powerful role of mitochondria is not limited to energy production. Mitochondria are also capable of determining survival of cells, which is often associated with mitochondrial dynamics. Mitochondrial dynamics consist mainly of fission, fusion, and cristae remodeling. Abnormal dysfunction in one of the three is often associated with a poor outcome. Pathological increases in mitochondrial fission are often accompanied by changes in the permeability of the outer cell membrane, which subsequently trigger a series of programmed cell deaths. However, a moderate increase in mitochondrial fission can be beneficial for axonal cells. For elimination of mitochondrial fusion, which is lethal to the cell and the organism, a moderate increase in fusion can contribute to cell survival and can repair damaged mitochondria to some extent. Remodeling of the cristae also plays an important role in the cell as they contain many energy-producing proteins and proteins that regulate cell death, such as cytochrome C. When the opening of the cristae expands, the contents of the cristae flow out, leading to a decrease in energy production and cell death.

The kidney is a very important body organ that serves as a “filter” of the body. The mitochondrial content of the kidney is quite high. Mitochondrial and kidney dysfunction caused by increased mitochondrial fission are complementary and form a vicious cycle. When the fission protein FIS1/DRP1 is reduced by drugs, it can have a protective effect on the kidney. Mitochondrial fission proteins undergo different post-translational modifications of proteins and often produce different fruitful effects, so a full understanding of their mechanisms of action is important for future prevention as well as treatment of kidney diseases.

## Figures and Tables

**Table 1:** Post-translational modifications of FIS1.

Type of Modification	Position	Upstream Molecules	Effects
Phosphorylation	Y38	MET	Phosphorylation
	/	DNA-PKcs	Phosphorylation
Ubiquitination	/	Parkin	Ubiquitination
	/	MARCH5	Ubiquitination
SUMOylation	K149	SENP3	DeSUMOylation
Acetylation	/	SIRT3	DeAcetylation

**Table 2:** Post-translational modifications of DRP1.

Type of Modification	Position	Upstream Molecules	Effects
Phosphorylation	Ser-616	Stat2	Phosphorylation
		PKCδ	Phosphorylation
		ERK1/2	Phosphorylation
		CaMKII	Phosphorylation
		Cdk5	Phosphorylation
		ROCK	Phosphorylation
		DUSP6	DePhosphorylation
	Ser-637	PKA	The specific role of S637 phosphorylation results from diverse internal and external parameters
		PKD	
		CaN	
		AMPK	
Ubiquitination	/	Parkin	Ubiquitination
	/	MARCH5	Ubiquitination
	/	SQSTM1/P62	Ubiquitination
SUMOylation	/	SENP3	DeSUMOylation

## Data Availability

Not applicable.
